# GMP Compliant Production of a Cryopreserved Adipose-Derived Stromal Cell Product for Feasible and Allogeneic Clinical Use

**DOI:** 10.1155/2022/4664917

**Published:** 2022-06-20

**Authors:** Mandana Haack-Sørensen, Ellen Mønsted Johansen, Lisbeth Drozd Højgaard, Jens Kastrup, Annette Ekblond

**Affiliations:** Cardiology Stem Cell Centre, The Centre for Cardiac, Vascular, Pulmonary and Infectious Diseases, Rigshospitalet Copenhagen University Hospital, 2100 Copenhagen, Denmark

## Abstract

The emerging field of advanced therapy medicinal products (ATMP) holds promise of treating a variety of diseases. Adipose-derived stromal cells (ASCs) are currently being marketed or tested as cell-based therapies in numerous clinical trials. To ensure safety and efficacy of treatments, high-quality products must be manufactured. A good manufacturing practice (GMP) compliant and consistent manufacturing process including validated quality control methods is critical. Product design and formulation are equally important to ensure clinical feasibility. Here, we present a GMP-compliant, xeno-free, and semiautomated manufacturing process and quality controls, used for large-scale production of a cryopreserved off-the-shelf ASC product and tested in several phase I and II allogeneic clinical applications.

## 1. Introduction

Advanced therapy (investigational) medicinal products (AT(I)MP), such as cell-based therapies with mesenchymal stromal cells (MSCs), including adipose-derived stromal cells (ASCs), are potential game changers for the field of regenerative medicine and immunosuppression [1–3]. In the last decade, numerous therapeutic strategies based on ASCs have been evaluated, and to date, more than one hundred clinical trials using ASCs have been registered in the database of the US National Institutes of Health (http://www.clinicaltrials.gov/).

ASCs are traditionally obtained from adipose tissue (AT) through minimally invasive procedures, such as water-assisted liposuction, followed by enzymatic or mechanical digestion of the lipoaspirate [4]. *In vitro* culture of the resulting stromal vascular fraction allows selection and expansion of colony forming ASCs, constituting >1% of seeded cells [5].

At the current stage of development, the shared challenge for the academic community is to translate and scale up research into applicable protocols compliant with good manufacturing practices (GMP). Furthermore, expectations to yield, safety, quality, efficacy, and feasibility should be met. Costs of cell products for late stage clinical trials must be realistic for marketing authorization applications [6]. GMP practices are designed to ensure that cell products are consistently produced and controlled according to specifications. Such practices are ensured through development of safe, reliable, and reproducible procedures, analyses, and tools and a robust expansion platform [3, 7–11]. The inherent biological variation and complexity in cell biology naturally makes this a challenging task. The technologies remain immature, supply chain and regulatory experience, and calls for ingenuity and risk analysis every step of the way. Risk analysis is dependent on existing knowledge about, e.g., origin of cells, expansion, and intended use, but is also continuously updated and nuanced to justify development, as knowledge increases.

There are many challenges to overcome when translating an academic protocol to a GMP compliant process. This includes the development of a quality management system, identification of safe and high-quality raw materials with a reliable supply chain, reduction of manual and open manipulation steps, qualification of equipment, validation of procedures and analyses for quality controls, a rapidly changing regulatory environment, and academic attainment of the regulatory mindset. On top of this, an eye is needed for feasibility of clinical use, which calls for development of off-the-shelf-products, and execution of stability studies, arranging distribution and procedures for clinical use.

Here, we present our approach for development of a scalable and quality upgradable GMP production platform for manufacturing of ASC products for clinical applications. The focal point is the expansion of cells in the functionally closed and semiautomated quantum cell expansion system, using human platelet lysate as a growth supplement [7, 12–16]. Moreover, we have validated and implemented quality control methods according to GMP or pharmacopoeial standards, and we have designed and formulated a cryopreserved off-the-shelf product, ready to use at the bedside after thawing, without further reconstitution. In other words, we describe GMP manufacturing of the allogeneic ASC-based and cryopreserved ATIMP, cardiology stem cell center_ASC (CSCC_ASC), as developed and used in our own national and international phase I and II clinical trials including patients with ischemic heart failure [17–19].

## 2. Materials and Methods

### 2.1. Process Overview

Cells were isolated from abdominal lipoaspirates from healthy donors and expanded for two-cell culture passages in closed, single-use bioreactors with hollow fibers, controlled by the quantum cell expansion system (Terumo BCT) (bioreactors). Stromal vascular fractions (SVF) were culture expanded to produce passage 0 (P0) cells (Intermediate cell product, ASC), which were further expanded in another passage to produce ASC P1 cells, constituting the active pharmaceutical ingredient in CSCC_ASC, the final cell product. Intermediate products and final products were cryopreserved and stored at<-180°C.

**Figure 1:**
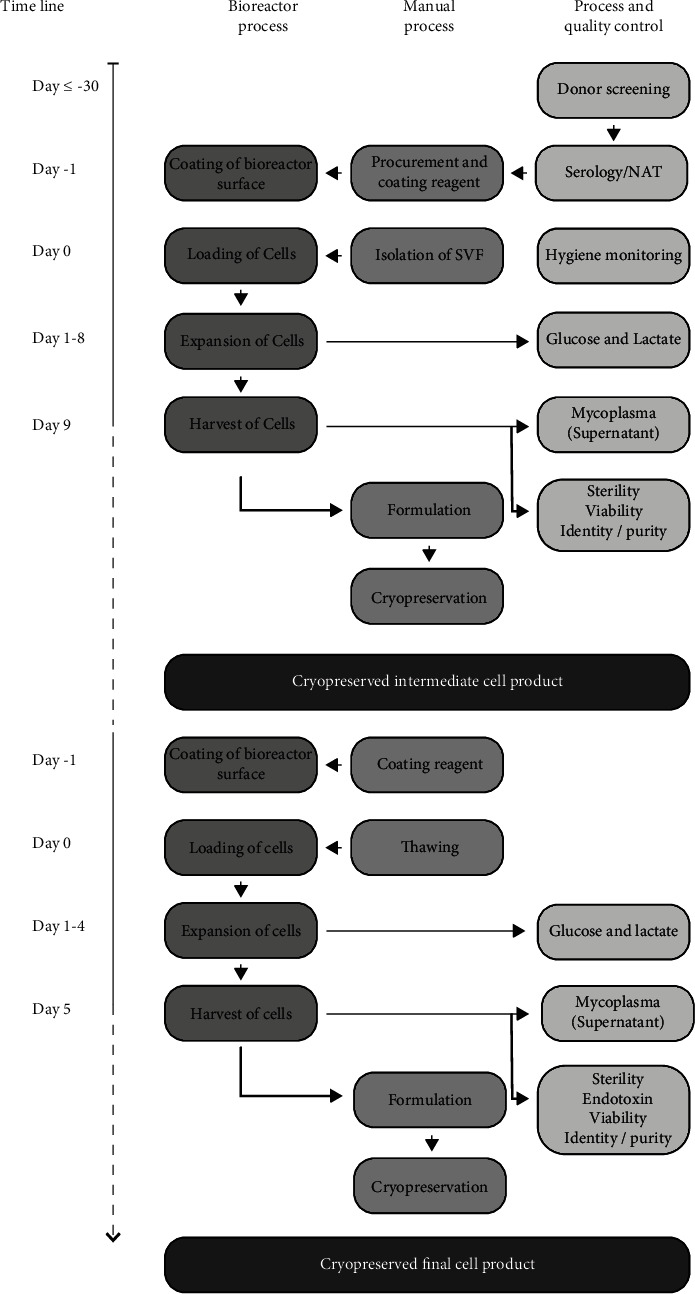
The manufacturing process overview. Timeline for different steps during manufacturing of intermediate and final cell product.

Manufacturing took place at Cardiology Stem Cell Centre (CSCC), which holds a manufacturing authorization for ATIMP production and quality control issued by the Danish Medicines Agency, and a tissue establishment authorization issued by the Danish Patient Safety Authority. The manufacturing procedure is in compliance with the European Guidelines for GMP for ATMP and for medicinal products for human use [20, 21] and European Tissue and Cells Directive (EUTCD) [22]. The process and product highlights of manufacturing are listed in Table 1, and the manufacturing process overview is illustrated in Figure 1.

### 2.2. Donor Selection

Seven donors (one male/six females), age 25-34 years and with BMIs between 27 and 35, were used in the production. Donor suitability was evaluated prior to procurement (liposuction) and was based on a health examination, a donor interview, a questionnaire, and screening for infectious disease markers, all according to EUTCD [22]. A donor was selected only if the screening concluded that the donor was healthy, free from risk factors, and the laboratory tests for infectious disease markers were negative. All donors signed an informed consent complying with the declaration of Helsinki.

Each donor was tested for HIV I/II, hepatitis B and C, syphilis, and HTLV I/II serology by serum analysis within 30 days prior to liposuction. In addition, a blood sample was drawn on the day of donation for repeated serology and NAT (nucleic acid testing) of HIV and hepatitis B and C. Donor testing was performed by the Virus Laboratory, Blood Bank, Department of Clinical Immunology, University Hospital Copenhagen Rigshospitalet, Denmark. Donor virus screening was performed with CE certified test systems only.

### 2.3. Starting Material

Liposuction was performed according to CSCC Standard Operating Procedures and in compliance with surgical procedures for sterile cosmetic surgery by trained plastic surgeons at Printzlau Private Hospital, Denmark. Liposuction of subcutaneous abdominal AT was performed under local anesthesia using water-assisted liposuction (WAL). The AT was harvested directly into a closed canister system and then transferred to a transportation canister without contact to other surfaces.

### 2.4. Lipoaspirate Preparation and SVF Isolation

The lipoaspirate was stored at CSCC at room temperature (RT) for <24 hr after procurement. SVF was isolated from the lipoaspirate by manual handling and enzymatic digestion, as previously described [12, 23]. In brief, each lipoaspirate was washed twice with 1x phosphate-buffered saline (PBS, -Ca^2+^ -Mg^2+^, and -phenol red) (Gibco, Life Technologies). The tissue was digested by GMP-grade collagenase NB6 (Nordmark, Germany) dissolved in Hank's Balanced Salt Solution (2 mM Ca^2+^) and neutralized with a complete medium. Complete medium, as used during the entire manufacturing process, contained minimum essential medium, MEM alpha (*α*MEM) without ribonucleosides and deoxyribonucleosides, (Gibco, Life Technologies), 1% penicillin/streptomycin (10,000 U/ml and 10,000 *μ*g/ml, respectively) (Gibco, Life Technologies), and 5% heparin-free human platelet lysate (hPL, custom made small batch, ≤16 donors, Sexton Biotechnologies). The SVF suspension was filtered, centrifuged, and resuspended in complete medium. Cell number and viability of cells in the isolated SVF was determined, using a NucleoCounter® NC-100™ (ChemoMetec, Denmark), according to manufacturer's instructions.

Hygiene monitoring with settle plates and contact plates (Oxoid, UK), used on bench top surfaces, operator gloves and garment was performed. Plates were analyzed by Clinical Microbiological Department, University Hospital Copenhagen Rigshospitalet, Denmark.

### 2.5. Cell Expansion

The bioreactors for culture expansion were placed in a clean room grade C [12, 13]. These were fed through two circulation loops with inlets for medium and reagents or cells. Waste was removed into a waste bag. The entire process allowed control of seeding, medium perfusion rate, harvest time, and medium washouts with predefined, customized, and automated settings (protocols). Samples of supernatants were drawn via a sterile connection device and a sterile barrier filter. Gas was provided to the systems as premixed supplies of 19.9% O_2_, 5% CO_2_, and 75.1% N_2_. The hollow fibers in the expansion sets for bioreactors were coated with cryoprecipitate (CPPT) (Blood Bank, Rigshospitalet, Denmark) to allow cells to adhere to the hollow fibers.

### 2.6. Preparation of Bioreactor

Bioreactors were prepared for cell load and expansion through mounting of single-use expansion sets, PBS, and media bags, followed by priming, coating, and conditioning of the mounted sets. Expansion sets were primed for coating by filling with PBS. Subsequently, PBS was replaced with CPPT. Coating settled for 20-24 hr, 37°C, and static conditions. Wash-out was done with complete medium to remove all residual CPPT, and the expansion set was conditioned to 37°C for at least one hour.

### 2.7. Loading of SVF into Bioreactors

The SVF diluted in 100 ml complete medium was loaded into the bioreactor for expansion of ASCs. The SVF was incubated for 24 h under static conditions, after which continuous feeding with medium was activated. Medium feeding rate started at 0.1 ml/min. On day 3, complete medium wash-out was performed to remove nonadherent SVF cells.

### 2.8. Loading of Intermediate Product into Bioreactors

One vial (50 × 10^6^ ASCs) of intermediate product was thawed in a 37°C water bath and diluted in complete medium. The cell suspension was loaded into one or two precoated bioreactors. Cells were incubated for 24 h under static conditions, after which continuous feeding with complete medium was activated. Medium feeding rate started at 0.1 ml/min.

### 2.9. Feeding and Harvest of ASCs

Based on glucose and lactate measurements of supernatants with a blood gas analyzer (ABL 835 FLEX, Radiometer), feeding rates were adjusted and growth rates predicted. Feeding rate was doubled upon predefined measures of lactate.

ASCs were harvested by loading and circulating 180 ml 1x TrypLE Select enzyme (Gibco, Life Technologies) into the system for 20 min, and the cell suspension was collected in the harvest bag of the closed quantum system.

### 2.10. Formulation, Filling, and Cryopreservation of Intermediate and Final Product

Harvested cells were transferred manually into 50 ml Falcon tubes in a grade A laminar air flow cabinet within a clean room grade B, washed with PBS, and counted with a NucleoCounter® NC-100™. Cells were resuspended in CryoStor10 (CS10) (Bio Life Solutions). Intermediate products were suspended with 10 × 10^6^ ASCs/ml in 5 ml CS10, whereas the final product CSCC_ASC was suspended with 22 × 10^6^ ASCs/ml (treatment dose = 110 × 10^6^ ASCs) in 5 ml CS10.

Sterile filling was performed manually under laminar air flow, at RT. 5 ml cell suspension was filled into CellSeal vials (Sexton Biotechnologies), and the vials were sealed with a CellSeal RF Sealing System (Vante). The integrity of correctly welded vials was validated by Sexton Biotechnologies. Immediately after sealing, each vial was stored on ice. Freezing was performed with an automated controlled rate freezer, Kryo 560-16 (Planer PLC). Next, vials were transferred on dry ice and stored in a CBS-v1500 Isothermal dry-storage system Nitrogen tank (Custom Biogenic Systems) at ≤ -180 C until clinical use (final product) or further expansion (intermediate product). This system and vial integrity minimize the risk of cross-contamination.

### 2.11. Raw Materials

A quality system ensured traceability of donors (starting material), and all critical raw materials used for the manufacturing of CSCC_ASC.

### 2.12. Raw Material Control and Considerations

Control upon reception was performed on all critical raw materials. Batch-specific documentation (quality certificates) was evaluated according to internal predefined specifications. Raw materials certified according to compendial methods were preferred, but not always available, and other quality standards (e.g., ISO) were accepted after a risk assessment [20, 24, 25].

Batch-specific documentation of sterility and pyrogenicity/endotoxin level of all raw materials was controlled. The absence of Mycoplasma was documented for all commercial materials of human origin. Each material was individually assessed; however, materials with pharmacopoeial standards were chosen where possible. High-risk raw materials for quality control, e.g., BACT/ALERT flasks for microbiological detection, and raw materials where batch-to-batch variation was expected (e.g., hPL) were qualified in-house prior to use in the GMP production.

### 2.13. Animal Origin

Viral safety, risk of xenogeneic immunoreactions, and risk of transmissible spongiform encephalopathy (TSE) and bovine spongiform encephalopathy (BSE) was accommodated by avoiding, where possible, raw materials where animal-derived constituents were used during the manufacturing process [26]. If this was not obtainable, products with a TSE certificate of suitability (CEP) from the EDQM were chosen.

### 2.14. Human Origin

Safety of raw materials of human origin (cryoprecipitate and hPL) was assured by choosing manufacturers that used donor selection and viral screening, according to current legislation for blood banks [22, 26–28], and for hPL, licensed by American Association of Blood Banks. Total viral load of potentially unknown viruses was contained for blood products, based on pooled donations by limiting the pool size < 16 donations [29–32].

### 2.15. Polymeric/Plastic Materials

The manufacturer's documentation of biocompatibility and cytotoxicity was considered for disposable polymeric/plastic materials and reagent containers.

Polymers may contain bovine resin, and manufacturer's documentation of TSE/BSE risk was evaluated for plastic materials.

### 2.16. Quality Control Methods

Compendial analytical methods were suitability tested, and noncompendial methods were either deemed appropriate for the current stage of development or validated, according to International Council for Harmonization of Technical Requirements for Pharmaceuticals for Human Use (ICH) guidelines [33], or guided by scientific assay validation protocols published by international societies.

In addition to quality controls for product specifications, decision-guiding process parameters were used, such as quality checks of all materials before production start, visual inspection of the starting material lipoaspirate and the digested AT, hygiene monitoring, measures of glucose and lactate upon which feeding rates were determined, and visual observation of harvested cell suspension for cell aggregates.

Quality controls were performed at different steps during manufacturing. Designated control points were donor screening, intermediate products, and final products. Intermediate products were tested for sterility, viability, and identity. The microbiological quality control included testing for bacteria, fungus, and Mycoplasma. Final products were tested for sterility, viability, identity, and purity. As microbiological quality control, bacteria, fungi, Mycoplasma, and endotoxin were tested. Release criteria for final products are listed in Table 2.

### 2.17. Cell Count and Viability

The SVF suspended in a complete medium, and newly harvested intermediate or final products suspended in PBS were counted, using a Nucleocounter NC100 device. Cell count and viability were determined according to the manufacturer's instructions.

### 2.18. Microbiological Testing

Sterility tests were conducted by an automated BACT/ALERT system (BioMérieux, France), suitability tested in accordance with Ph.Eur. [34, 35]. The residual volume, 1% (≥2 × 100 *μ*l) of each batch after aliquoting into cryovials, was used for testing, and each sample was inoculated in to BACT/ALERT ® iFA Plus (aerobic) and BACT/ALERT ® iFN Plus (anaerobic) flasks (both from BioMérieux, France).

Samples were incubated at 34.5-35.5°C for 12 days. If positive, species were identified by Clinical Microbiological Department, University Hospital Copenhagen Rigshospitalet, Denmark.

### 2.19. Endotoxin Test

The presence of bacterial endotoxins was tested on the residual volume of each batch of final product after aliquoting into cryovials. Testing was performed at CSCC with the compendial method LAL kinetic chromogenic method D, using the Endosafe-PTS system (Charles River, USA), according to the manufacturer's instructions. The protocol was validated according to Ph. Eur [36, 37], which included a test for interfering factors and a suitability test. Acceptance criteria for endotoxin was 70 IU/ml = Endotoxin Limit = *K* (threshold pyrogenic dose/kg body mass)/*M* (maximum recommended bolus dose).

### 2.20. Mycoplasma

Mycoplasma testing was performed with a nucleic acid amplification technique (NAT) by Eurofins Pharma Biolab, Italy, according to a validated protocol [38]. The presence of Mycoplasma was tested on inner loop supernatants from all bioreactor expansions immediately prior to cell harvest.

### 2.21. Identity and Purity

Flow cytometry was used to identify the active ingredient, i.e. the ASCs (CD90^+^, CD73^+^, and CD105^+^) and determine the purity (impurities defined as CD45- and HLA-DR-positive cells) of the intermediate product and the final product CSCC_ASC prior to freezing. Upon cell harvest, the cells were washed in PBS and stained with FVS780 (BD) for 10 min at RT in the dark. Cells were washed in fluorescence-activated cell sorting PBS (FACS-PBS) (Hospital Pharmacy), 1% ethylenediaminetetraacetic acid (EDTA) (Hospital Pharmacy), supplemented with 10% *γ*-irradiated and heat-inactivated fetal calf serum (FBS) (Gibco, Life Technologies) and centrifuged at 300 G for 5 min at RT. The cells were stained for 20-30 min at RT in the dark with the following antibodies in separate tubes: CD73-APC (AD2), CD90-APC (5E10), CD105-PerCP-Cy.5.5 (266), CD45-PE (HI30), and HLA-DR-PE (G46-6); washed in FACS-PBS and centrifuged. Subsequently, the cells were resuspended in PBS and acquired on a Navios IVD flow cytometer (Beckman Coulter). Data were analyzed using Kaluza software (Beckman Coulter) based on 10000 single and live cells. The protocol was validated by CSCC, according to European Pharmacopoeia and International Council of Harmonization [33, 39]. Acceptance criteria are listed in Table 2.

### 2.22. In-Use Stability

The final product was thawed, as prior to clinical use. Next, the cells were left at RT for either 1/2, 1, 2, or 3 hours to simulate delay at the bed side. Viability of the cells were measured, and 1 × 10^6^ cells from each time point were seeded in T75 flasks (Thermo Fisher Scientific) with complete medium and incubated at 37°C for 48 h to calculate population doublings (PDs). For acceptance criteria, see Table 2 (identity and purity were only evaluated at 0 hour).

### 2.23. Storage Stability

Product stability during storage (nitrogen dry storage < −180°C) must be continuously monitored [20, 21]. Therefore, measures of viability, identity/purity, sterility, and PDs were evaluated. Performance of thawed cells was investigated according to acceptance criteria: population doubling (PD) > 1; viability > 80% and identity/purity: CD90, CD105, *CD*73 > 80%; CD45 < 3%; and HLA-DR < 5%, at base line (0 months) and after 12 and 24 months of storage.

## 3. Results

### 3.1. SVF

**Figure 2:**
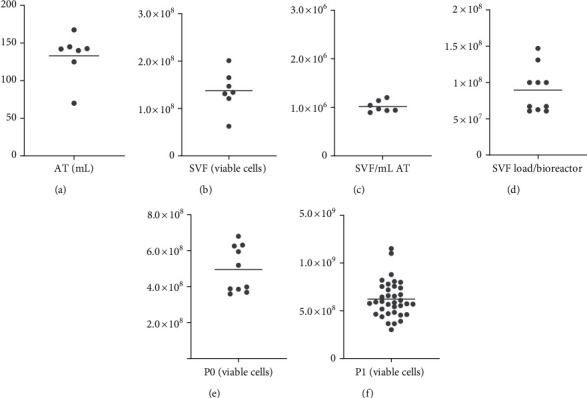
Overview of yield. (a) Volume of adipose tissue (AT) processed per donor (*n* = 7). (b) Number of stromal vascular fraction (SVF) isolated from AT (*n* = 7). (c) Yield SVF per ml AT. (d) Number of SVF loaded into the bioreactor (*n* = 10). (e) ASC P0 harvested per batch (n = 10). (f) ASC P1 harvested per batch (*n* = 36). The line represents the mean value.

The starting material (lipoaspirate) was procured from seven healthy volunteer donors. Mean volume AT processed was 133 ml (70-167 ml) (Figure 2(a)). The SVF mean yield was 1.37 × 10^8^ cells (6.26 × 10^7^–2.01 × 10^8^) (Figure 2(b)), which corresponded to an average of 1.02 × 10^6^ ± 4.00 × 10^4^ SVF per ml AT (Figure 2(c)) with a mean cell viability of 83% (72%-90%, data not shown).

### 3.2. Passage 0, P0

For the first culture expansion, in average 9.60 × 10^7^ SVF (6.07 × 10^7^–1.47 × 10^8^) were loaded into one or two bioreactors (Figure 2(d)). After 8-10 days of expansion, in average 4.95 × 10^8^ (3.68 × 10^8^–6.80 × 10^8^) viable ASCs were harvested per bioreactor (Figure 2(e)), with a mean cell viability of 89% (85%-95%, data not shown). This resulted in a minimum of 7 and a maximum of 23 vials of intermediate products per donor.

### 3.3. Passage 1, P1

To produce the final product, 25 × 10^6^ or 50 × 10^6^ ASCs from intermediate products, were loaded into a bioreactor. One batch was defined as a final expansion (P1) in one closed disposable bioreactor. Batch size was the total number of cells obtained upon harvest. Mean viable cell yield obtained from 36 batches was 6.02×10^8^ (3.01×10^8^–1.15 × 10^9^) (Figure 2(f)). A minimum of two and a maximum of nine vials with 110 million cells were obtained per batch.

### 3.4. Identity and Purity

**Figure 3:**
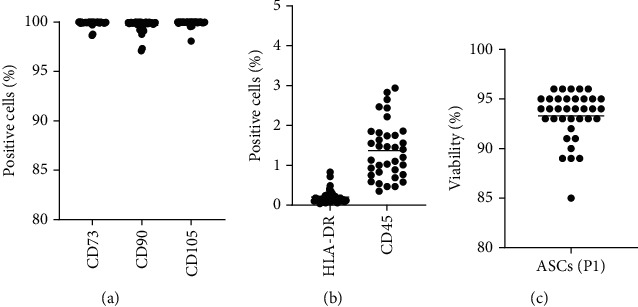
Identity, purity, and viability. (a) % expression of positive surface markers. (b) % expression of negative surface markers. (c) Viability of CSCC_ASC (*n* = 36). The line represents the mean value.

The identity and purity of cells of 36 batches of final product, derived from seven donors, were quality controlled by flow cytometry, according to the CSCC validated protocol. Debris, doublets, and dead cells were excluded from the analysis, and the expression of the stable positive ASC markers CD73, CD90, and CD105 were further examined, as a measure of identification of the active ingredient (Figure 3(a)). All markers were present on at least 97% of the live single cells in each the batch, thus meeting the acceptance criteria at >80%. Also, the antigen-presenting marker HLA-DR and the stable negative ASC marker CD45 were examined, as a measure of impurity and hence an indirect measure of purity (Figure 3(b)). The impurity spanned from 0.04 to 0.83% positive cells for HLA-DR and 0.35 to 2.94% positive cells for CD45, respectively. All 36 batches met CSCC acceptance criteria (see Table 2).

### 3.5. Sterility

All the produced intermediate and final product batches were sterile, and no Mycoplasma or bacterial endotoxin were detected (data not shown).

### 3.6. Viability

All batches of final product met the acceptance criteria of a viability above 80%, with a mean cell viability of 93% (89%-96%) (Figure 3(c)).

### 3.7. Success Rate

Production success rate for release of the final products was 92%. No batches were rejected, based on failure to comply with acceptance criteria. Three batches out of the 36 batches produced were excluded due to other observations. We observed cell aggregates in two batches (from the same intermediate product) after harvest. One batch was discarded during the washing steps after harvest, due to a damaged utensil.

### 3.8. In-Use Stability of CSCC_ASC

**Figure 4:**
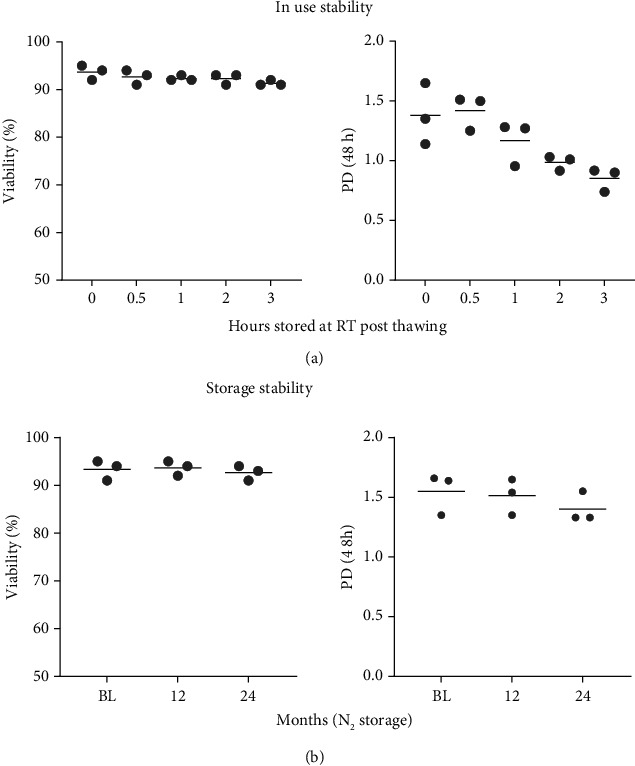
In use and storage stability. (a) In-use stability of thawed cells at room temperature (RT) within three hours. (b) Long-term stability of nitrogen stored CSCC_ASC over 24 months. PD: population doublings; BL: baseline. The line represents the mean value.

In-use stability of the final product was tested on three batches (based on three different donors). The viability of the cells immediately after thawing (0 hr) was 92%-95%. The viability remained above the accepted 80% live cells after storage for three hours at RT 91-92% (Figure 4(a)). Also, the ability to proliferate was investigated. The PD of cells left at RT for 0 h and grown for 48 h in culture was 1.14-1.65 and cells that had been left at RT for 1/2 and 1 hr still had PDs above one, meeting the acceptance criteria that all batches had PD > 1. However, cells that had been left at RT for two and three hours had a PD below one (0.92-1.03 and 0.74-0.92, respectively), hence not meeting the acceptance criterium.

### 3.9. Storage Long-Term Stability of CSCC_ASC

A long-term stability program was implemented for CSCC_ASC from three batches (based on three different donors) stored for 12 and 24 months at -180°C, compared with cells stored for ≤1 month (baseline (BL)). Stability was determined based on sterility, viability, identity/purity, and function (proliferation).

All vials were sterile, and the identity and purity were unchanged after storage, compared to BL (data not shown). Immediately after thawing (BL), the viability of the cells was 91-95% (Figure 4(b)). The viability remained above 80% after 12 or 24 months of storage, 92-95% and 91-94%, respectively. The PD at BL over 48 h was 1.35-1.66. After 12 and 24 months, the PD remained above 1 1.35-1.65 and 1.33-1.55, respectively. The stability study showed that after storage for 24 months at -180°C the cells still met the acceptance criteria set forth in Table 2.

## 4. Discussion

Application of MSCs has emerged as a promising tool for regenerative medicine, as the efficacy of MSC-based cell therapy has been demonstrated for a broad spectrum of diseases [2, 40]. ASCs have gained attention, as they can be isolated from AT at a relatively high yield and expanded extensively *in vitro*. The prerequisite for successful ASC-based therapies is however the development of safe, reliable, high-quality, and scalable manufacturing processes that can provide affordable cell products for feasible treatment regimens in compliance with both good manufacturing, distribution, and clinical practice (GMP, GDP, and GCP, respectively).

With this in mind, we implemented a semiautomated expansion platform, for production of a cryopreserved, high-concentrated, off-the-shelf ASC product, suited for allogeneic clinical use. The investigational product developed is xeno-free, has proven 24 months stability, and has been designed for clinical feasibility and dissemination of treatment.

The manufacturing technology described builds on an in-house history of manual flask-based expansion of MSCs, from both the bone marrow and adipose tissue, for autologous clinical treatment in ischemic heart disease patients. Manual protocols provided autologous cells for three clinical studies [41–43]. The use of manually expanded, fresh autologous MSCs required weeks of open procedures and several labor-intensive manual steps. Quality control was challenging due to the short storage of the fresh cell product. The result was a nonstandardized product and the dosage given for each patient varied immensely.

Thus, a cryopreserved allogeneic product was developed. This minimized donor variability, patient discomfort, and healthy young donors could be chosen to minimize the influence of patient comorbidities on product quality. Nitrogen dry storage (<-180°C) made quality control and distribution more feasible, as well as the logistics of the treatment.

Using allogeneic donor tissue as a starting material allows uniformity and control of factors, such as donor age, body mass index and health status. Donor-related factors with potential effects on cell yield and *in vitro* functions are numerous [44–46]. Studies have shown differences between tissue donors with comorbidities and healthy controls, e.g., ASCs from obese subjects and type-2 diabetics presented decreased immunosuppressive capabilities [47], and ASCs from obese donors revealed a lower angiogenic potential, compared with lean controls [48, 49]. Differences have also been observed in the metabolism of ASCs from lean and obese subjects [50, 51].

To further improve consistency of cell products, to minimize contamination risks, and to improve production yield, we implemented closed and automated cell expansion with the quantum cell expansion system. The bioreactor system significantly reduces operative variability, time, and costs, compared to traditional flask-based techniques [6, 12, 52].

To produce ASCs in large-scale and meet clinical demands, identification of a safe and efficient growth supplement was as crucial as the use of bioreactors. Hence, we introduced the use of hPL, as a safe and efficient alternative to FBS [12, 13, 53]. To avoid gel formation and a potential heparin mediated damage to the CPPT coating, a fibrinogen depleted version of hPL was chosen. Furthermore, hPL contains growth promoting factors that significantly promote cell growth over FBS [13, 53, 54]. Using human blood-derived raw materials for manufacturing of ATIMPs, pathogen reduction of materials or consideration of pool size is necessary. As pathogen-reduced versions were not available in 2015, when this platform was designed and approved for clinical use, we considered the pool size, i.e., the number of blood units pooled per batch of the raw material used. Hence, custom made small batch hPL with batch sizes ≤ 16 donors were used, to contain the risk of transfer of unknown pathogens. Small batch blood-derived raw materials do however exhibit high variation between batches, due to the biological nature of the material. An increased pool size would have reduced this inherent variation, and thereby improved standardization of our ATIMP manufacturing process [26, 29]. Since we initiated the production, pathogen-reduced large batch hPL, with a shared impact on safety and quality, has become commercially available (e.g., nLiven PRTM, Sexton Biotechnologies). Hence, CSCC has discontinued the use of small batch hPL and performed a comparability exercise to evaluate quality, efficacy, and safety of pathogen reduced hPL [55–57].

A recurring challenge in ATIMP manufacturing is the sparse availability of suitable, affordable GMP compliant raw materials. This calls for a thorough evaluation, risk analysis, and selection of the best and most suitable raw materials available prior to introducing them to production. Obtaining full insight into origin, TSE safety, process-related impurities, and reduction thereof, acceptance levels, and quality systems used, is a time-consuming and costly task that much awaits market and regulatory initiatives.

Publication of official European standards for GMP production and adaptation of reference works for quality control of ATMPs are still in the making. European Commission published Good Manufacturing Practice Guidelines on Good Manufacturing Practice specific to Advanced Therapy Medicinal Products in 2017, and the European Pharmacopoeia implemented the General Chapter 5.2.12. Raw materials of biological origin for the production of cell-based and gene therapy medicinal products, also in 2017. Much needed guidance to ATIMP developers, but with this project as an example (initiated in 2015), it also illustrates how ATIMP development is in constant need of adaptation, since products and guidelines are designed in parallel.

To guarantee dissemination of clinical therapy, feasibility of the treatment concept is key [58]. CSCC_ASC is formulated as a cryopreserved ready-to-use allogeneic cellular product, that advance ASC therapies to ‘off-the shelf' approaches. This allows the cell product to be available in a timely fashion under acute and chronic clinical settings.

Stability studies are required for prolonged storage to ensure efficacy upon use. Hence, sterility, phenotype, viability, and proliferation potential of cells after storage was determined. Viability of cells measured immediately after thawing should be interpreted with caution, as it does not account for delayed onset death. Delayed onset death manifests itself postthaw over hours and days *in vitro*, through apoptosis and necrosis [59]. As such, regrowth of the thawed cell population is a more credible measure of stability than immediate viability itself and a valuable parameter for decision-making. As the prevailing view on ASC efficacy is that a multitude of biological mechanisms work in concert with the actual *in vivo* environment met upon injection, basic *in vitro* function of cells, like proliferation, is a plausible first premise for potency, and hence a very first predictor of clinical efficacy, during early clinical development [60].

As development progresses, prediction of efficacy of the cell product is nuanced by identification of specific modes and mechanisms of action [61, 62], and development of quality controls validated to meet requirements for standard QC assays matures [63]. Reaching the stage of pivotal clinical trials, validated potency assays must be included in the quality control.

CSCC_ASC has proven safe in a first-in-man clinical trial with direct intramyocardial injections in ischemic heart disease patients (EudraCT no.: 2014-002980-13). A six-month follow-up has proven no serious or nonserious adverse events related to CSCC_ASC. Chromosomal stability of CSCC_ASC batches made for the safety study was analyzed with comparative genomic hybridization, and no imbalanced chromosomal rearrangements were detected [19].

Furthermore, CSCC_ASC has proven safe-to-date in a phase I safety trial with nonischemic dilated cardiomyopathy (EudraCT no.: 2018-002538-19) and two phase II double-blinded randomized clinical trials with ischemic heart disease (EudraCT No: 2015-002929-19 and 2015-01560-19) [17, 18]. Trials have currently included >200 patients, and no product-related adverse events have been described to date.

## 5. Conclusion

We have developed a feasible, semiautomated, GMP, GDP, and GCP applicable platform for manufacturing, quality control, and storage of an allogeneic cryopreserved off-the-shelf adipose tissue-derived stem cell product. The product has proven safe in multiple clinical trials, and customized versions are potentially applicable for numerous clinical indications. Knowledge, possibilities, and expectations to manufacturing of cell therapy products are constantly evolving. Regulatory guidelines, raw materials, process knowledge, and validated analytical tools have emerged since the initiation of this project, leaving space for further development of the platform. The GMP manufacturing platform described, however, constitutes a solid foundation upon which such development and quality upgrades can be based, as cell therapies reach pivotal trials.

## Figures and Tables

**Table 1:** Process steps in the production of the ATIMP for allogeneic clinical application.

Processes steps	Highlights
Donor selection	Young healthy volunteer donors (male and female)
Donor serology/NAT	HIV, hepatitis B and C, syphilis, and HTLV I/II
Procurement	Water assisted liposuction
Starting material (tissue)	Lipoaspirate (abdominal subcutaneous adipose tissue)
Raw materials	Highest grade available
Isolation of SVF	Manual enzymatic digestion
Cell expansion	Closed and semi-automated
Culture conditions	Dynamic and xeno-free
Formulation (excipient)	Cryopreservation media
Quality control methods	According to product specifications
Storage	Liquid nitrogen dry storage
Stability	Storage for two years documented
Clinical use	Allogeneic application

**Table 2:** Release criteria.

Attribute	Method of analysis	Acceptance criteria
Donor screening	Serology (antibody testing)	Negative for anti-HIV1, 2 anti-HCV, HBsAg, anti-HBc, syphilis, HTLV I/II
	Nucleic acid amplification technique (NAT, antigen testing)	Negative for HIV, HBV, and HCV

Sterility	Bacteria/fungus: BACT/ALERT microbial detection system (aerobic and anaerobic)	Negative/negative
	Bacterial endotoxin (LAL, method D)	<70 IU/ml
	Mycoplasma (NAT)	Negative

Identity and purity	Flow cytometry	CD90 > 80%
		CD105 > 80%
		CD73 > 80%
		CD 45 < 3%
		HLA-DR < 5%

ASC viability	Nucleocounter NC100	>80%

## Data Availability

All data generated to support the findings of this study are included within the article.
